# Spontaneous rupture of the plantar fascia: a case report

**DOI:** 10.3389/fresc.2024.1470002

**Published:** 2024-08-27

**Authors:** Michele Venosa, Emilio Romanini, Lorenzo Vitale, Giandomenico Logroscino

**Affiliations:** ^1^ RomaPro Center for Hip and Knee Arthroplasty, Polo Sanitario San Feliciano, Rome, Italy; ^2^ GLOBE, Italian Working Group on Evidence Based Orthopaedics, Rome, Italy; ^3^ Department of Life, Health and Environmental Sciences, University of L'Aquila, L’Aquila, Italy; ^4^ Polo Sanitario San UOSD, Department of Mini-Invasive and Computer-Assisting Orthopaedic Surgery, San Salvatore Hospital, L'Aquila, Italy

**Keywords:** plantar fascia, plantar fascia rupture, physical therapy, rehabilitation, case report

## Abstract

**Introduction:**

The rupture of the plantar fascia is a rare but significant injury that predominantly affects athletes and individuals engaged in high-impact activities. Sudden increases in physical activity, direct trauma, corticosteroid injections, and chronic degeneration from plantar fasciitis can predispose individuals to rupture. It can involve a complete or partial tear of the plantar fascia fibers, leading to a loss of structural integrity and functional support. The tear may occur at the origin, mid-portion, or insertion of the fascia. Spontaneous ruptures of the plantar fascia (occurring without any predisposing factors) are rarely observed in clinical practice. No guidelines or other unequivocal recommendations are available for this pathological condition.

**Method:**

A healthy 35-year-old male who works in an office setting and is a recreational cyclist with a silent clinical anamnesis experienced a spontaneous rupture of the plantar fascia of the left foot with no history of trauma. He exhibited significant localized tenderness and swelling in the medial arch of the left foot with difficulty bearing weight on the affected foot. An MRI confirmed a partial rupture of the medial cord of the plantar fascia accompanied by surrounding inflammation. The patient underwent conservative treatment, which included rest, immobilization, physiotherapy (ultrasound therapy, high-power laser therapy, and transcutaneous electrical nerve stimulation), rehabilitation, and a gradual return to activity.

**Results:**

At the 12-week follow-up, the patient reported a significant reduction in pain and marked improvement in functional mobility (as confirmed by VAS and Foot Function Index scores). Physical examination showed no tenderness, and the patient could bear full weight on the foot without discomfort. A follow-up ultrasound demonstrated complete resolution of the plantar fascia rupture and no residual inflammation.

**Discussion:**

This case underscores the effectiveness of an integrated rehabilitative approach and provides a framework for managing similar cases in clinical practice.

## 1 Introduction

The plantar fascia, also known as the plantar aponeurosis, is a thick, fibrous band of connective tissue that originates from the medial tubercle of the calcaneus (heel bone) and extends distally to the proximal phalanges of the toes (1). It is composed of three distinct segments: the medial, central, and lateral bands. The central band is the thickest and most significant portion, providing the majority of the structural support. It spans the length of the foot and divides into five slips that insert into the base of the proximal phalanges. Each slip is connected to the flexor tendons, sheaths, and the superficial transverse metatarsal ligament. The medial band is the thinnest and least distinct of the three. It extends from the medial aspect of the calcaneus and integrates with the fascia covering the abductor hallucis muscle. The lateral band extends from the lateral aspect of the calcaneus and integrates with the fascia overlying the abductor digiti minimi muscle. The medial and lateral bands, though thinner, also contribute to the overall function and integrity of the plantar fascia (2). The primary function of the plantar fascia is to provide structural support to the arch of the foot, contributing to both static and dynamic stability, and preventing flattening under the weight-bearing conditions. It plays a crucial role in the biomechanics of the foot, particularly during the gait cycle (3, 4). It acts as a shock absorber, distributing the impact forces during activities such as walking, running, and jumping (5). During the toe-off phase of the gait cycle, the plantar fascia tightens as the toes dorsiflex (6). This tension elevates the arch and provides a rigid lever for propulsion, known as the windlass mechanism (3). This mechanism enhances the efficiency of the foot during push-off.

Pathological conditions of the plantar fascia often arise from repetitive stress, overuse, or acute trauma. Plantar fasciitis is a common condition characterized by inflammation of the plantar fascia, typically at its origin on the calcaneus. It is often a result of overuse, improper footwear, or biomechanical abnormalities (7). Patients with plantar fasciitis typically present with heel pain, especially with the first steps in the morning or after prolonged periods of inactivity. The pain is often localized to the medial aspect of the heel and may decrease with activity. The underlying pathology involves microtears and degeneration of the collagen fibers within the plantar fascia, leading to inflammation and thickening of the fascia. Chronic cases may exhibit neovascularization and fibrosis (8). Plantar fascia rupture is a less common but more severe condition, which can generally occur as a complication of chronic plantar fasciitis. Sudden increases in physical activity, direct trauma, corticosteroid injections, and chronic degeneration from plantar fasciitis can predispose individuals to rupture (9). Patients with a plantar fascia rupture often report a sudden, sharp pain in the arch or heel of the foot, sometimes accompanied by a popping sound. Swelling, bruising, and an inability to bear weight are common findings. A rupture involves a complete or partial tear of the plantar fascia fibers, leading to a loss of structural integrity and functional support. The tear may occur at the origin, mid-portion, or insertion of the fascia (10). Spontaneous rupture of the plantar fascia is a rare and uncommon yet impactful injury characterized by the sudden tearing of the fibrous tissue along the bottom of the foot. It can occur without any predisposing factors, in healthy patients engaging in moderate low-impact activities. The management of plantar fascia ruptures can be challenging, since there is no unequivocal strategy, with treatment options ranging from conservative approaches to surgical intervention. Despite the potential benefits of surgery, conservative management is often preferred due to its non-invasive nature and favorable outcomes (11).

This case report details an integrated rehabilitative approach to a spontaneous plantar fascia rupture in a healthy 35-year-old male. By documenting the clinical presentation, diagnostic process, treatment plan, and outcomes, this report aims to provide insight into the effectiveness of non-surgical management strategies and contribute to the existing literature on this rare condition.

## 2 Methods

### 2.1 Case presentation

A 35-year-old male with a BMI of 24.2, who works in an office setting and is a recreational cyclist (riding about 80 km on asphalt twice a week), presented with a sudden onset of sharp pain in the arch of his left foot while walking (VAS score: 8). He reported no episodes of plantar fasciitis over the last years or previous trauma to the feet or lower extremities. He denied any remarkable pre-existing or concomitant systemic illnesses. He hasn't taken anti-inflammatory, corticosteroid, or antibiotic therapy in the last year. He had practiced soccer, archery, and swimming recreationally only during his childhood and adolescence. He routinary wears loafers when he's at work and sneakers in his recreational time. He is not aware of previous pathological conditions of the plantar fascia or relevant genetic abnormalities in his family.

### 2.2 Clinical examination

**Figure 1:**
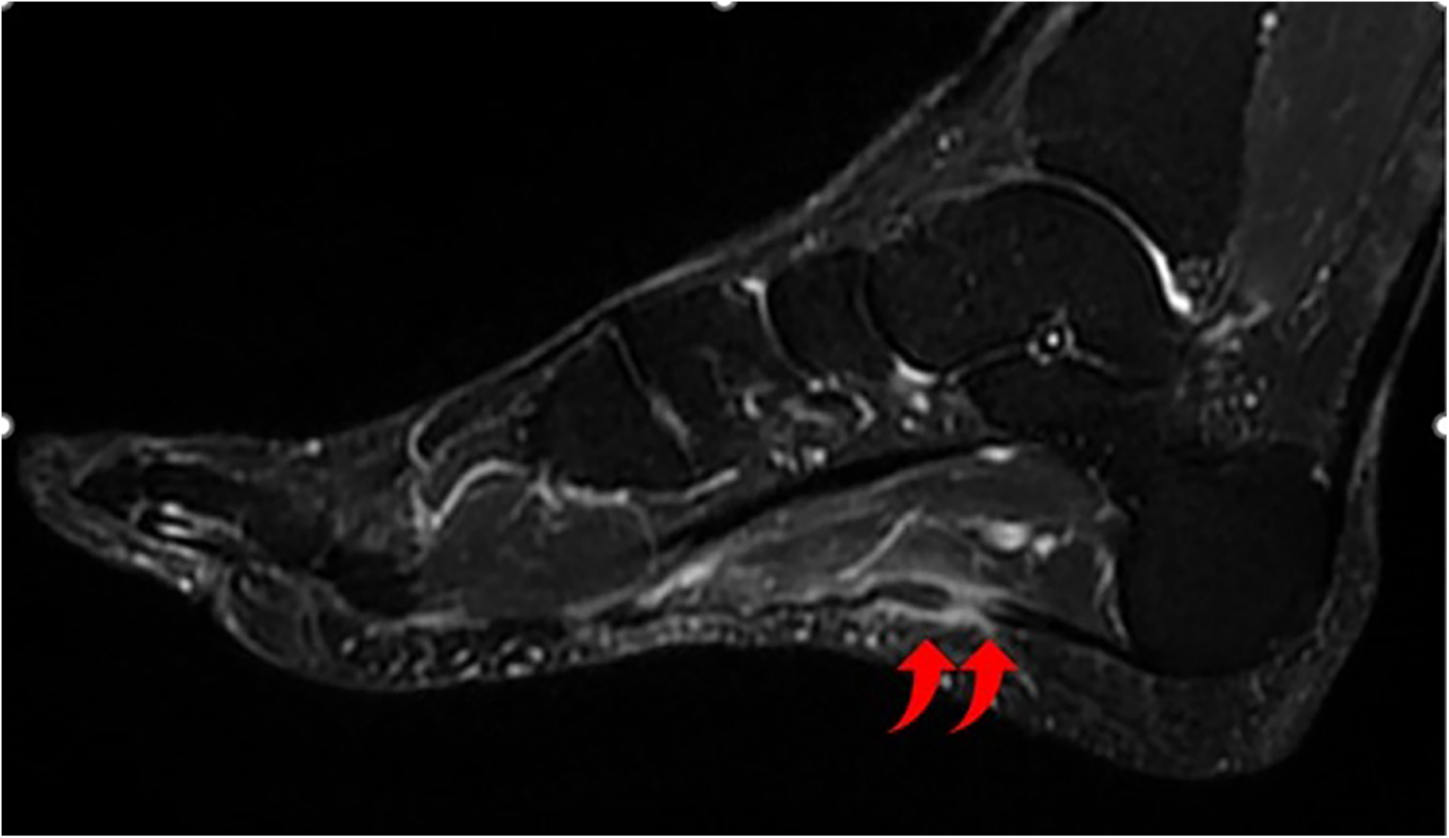
T2-weighted MRI image showing the disruption of the medial cord of the plantar fascia at its proximal third with edema of the plantar fascia and surrounding soft tissue.

During the initial examination, the patient exhibited significant localized tenderness and swelling in the medial arch of the left foot. He had difficulty bearing weight on the affected foot. We assessed the continuity and tension of the plantar fascia during passive dorsiflexion of the ankle and metatarsophalangeal (MTP) joints and then compared these findings with those of the opposite side. Notably, a palpable gap was detected in the plantar fascia, raising suspicion of a rupture. There were no visible deformities or ecchymosis. The pain was exacerbated by the dorsiflexion of the first metatarsophalangeal joint; no significant clinical findings were detected in other areas of the foot and ankle. Differential diagnoses considered included acute plantar fasciitis, stress fractures of the calcaneus or metatarsals, and tendon pathology. Given the clinical presentation, an MRI was performed, which confirmed a partial rupture of the medial cord of the plantar fascia accompanied by surrounding inflammation (Figure 1). Foot Function Index (FFI) was administered at this time to measure the impact of this pathological condition in terms of pain, disability, and activity restriction (FFI score: 62%).

### 2.3 Management protocol

The patient was managed conservatively through a phased approach: in the first two weeks, he was advised to completely avoid weight-bearing activities, walking with the aid of two crutches. A short-leg cast was applied to immobilize the foot and promote healing. Ice application was recommended to reduce swelling and provide pain relief. Nonsteroidal anti-inflammatory drugs (NSAIDs) were prescribed to alleviate pain and control inflammation. For this reason, the patient was instructed to remove the cast only to allow the ice application (15 min; 6 times a day for two weeks), preventing any active or passive strain on the plantar arch. An analgesic instrumental physiotherapeutic program began immediately, by performing a session three times a week, and continued for 4 weeks. The physical therapy included a combination of ultrasound therapy (UT) and high-power laser therapy (HPLT) FP3® once a week and a combination of UT and transcutaneous electrical nerve stimulation (TENS) twice a week (Table 1). After two weeks the patient removed the cast, allowing partial weight-bearing with two crutches, with limited range of motion of the ankle and limited strain on the plantar arch, to gradually reintroduce stress to the healing tissue. At 6 weeks full weight-bearing was allowed and gentle stretching exercises targeting the Achilles tendon and plantar fascia were initiated (Table 2). Low-impact activities, such as swimming and cycling, were encouraged to maintain cardiovascular fitness without placing undue stress on the foot. Progressive resistance exercises were introduced to strengthen the intrinsic and extrinsic muscles of the foot. Proprioceptive training was performed to correct any abnormal gait patterns that had developed due to the injury (lack of fluency in the walking scheme with an unconscious tendency to increase the weight on the lateral side of the foot). A gradual return to weight-bearing activities was implemented, with an emphasis on using proper footwear and orthotic support to prevent recurrence. A summary timeline of our integrated rehabilitative approach is reported in Table 3.

**Table 1:** Physical therapy protocol–3 sessions/week started immediately after the diagnosis and performed for 4 weeks.

Physical therapy (instrumentation)	Therapeutic protocol
UT	1.5 Watt/cm^2^–3 MHz–20 min–3 sessions/week
HPLT	*λ*: 780–1,100 nm–10 Watt–120 J/cm^2^–8 min–1 session/week
TENS	100 μs–70 Hz–20 min–2 sessions/week

**Table 2:** Achilles tendon and plantar fascia stretching exercises scheduled for the patient.

Phase	Exercises	Details	Frequency
Phase 1 (this phase begins 6 weeks after the diagnosis)	Toe curls with a towel	Scrunch a towel with your toes while seated.	2 sets of 10 reps, 2–3 times/day
	Seated plantar fascia stretch	Pull toes back toward the shin while seated.	Hold 20–30 s, 3 sets, 3 times/day
	Achilles tendon stretch (seated)	Use a towel to pull your toes toward you with your knee straight.	Hold 20–30 s, 3 sets, 3 times/day
Phase 2 (this phase begins 8 weeks after the diagnosis)	Standing calf stretch (gastrocnemius)	Stand facing a wall, back leg straight, heel on the ground.	Hold 20–30 s, 3 sets, 3 times/day
	Standing soleus stretch	Slightly bend back the knee while keeping the heel down.	Hold 20–30 s, 3 sets, 3 times/day
	Plantar fascia stretch using a step	Lower heel below step level while standing on the edge of the step.	Hold 20–30 s, 3 sets, 2–3 times/day
	Toe extension stretch	Pull the toes back with a hand to stretch the plantar fascia	Hold 20–30 s, 3 sets, 2–3 times/day
Phase 3 (this phase begins 10 weeks after the diagnosis)	Calf raises	Stand on the edge of a step, and raise and lower your heels.	2–3 sets of 10–15 reps, once daily
	Dynamic stretching (lunges with Achilles stretch)	Perform lunges while keeping back your heel on the ground.	10 reps each leg, 2 sets, once daily
	Towel stretch with resistance band	Use a resistance band to stretch Achilles and plantar fascia.	Hold 30 s, 3 sets, twice daily
	Tennis ball roll	Roll a tennis ball under your foot for massage/stretch.	2–3 min, 2–3 times/day
Phase 4 (this phase begins 12 weeks after the diagnosis)	Daily stretching routine	Continue all previous stretches to maintain flexibility.	Daily

**Table 3:** Timeline of our conservative plan for the spontaneous rupture of the plantar fascia.

0–2 weeks	- No weight-bearing activities and walking with two crutches - Short-leg cast immobilization - Ice applications (15 min; 6 times a day) - NSAIDs - UT + HPLT + TENS (3 physiotherapeutic sessions/week)
2–4 weeks	- Partial weight-bearing and walking with two crutches (limited strain on the plantar arch) - UT + HPLT + TENS (3 physiotherapeutic sessions/week)
4–6 weeks	- Progressive weight-bearing and walking with two crutches (limited strain on the plantar arch)
6–8 weeks	- Full weight-bearing without crutches - Achilles tendon and plantar fascia stretching exercises - Low impact activities
8–12 weeks	- Achilles tendon and plantar fascia stretching exercises - Progressive resistance exercises - Proprioceptive training - Low impact activities

## 3 Results

At the 12-week follow-up, the patient reported a significant reduction in pain (VAS score: 2) and marked improvement in functional mobility (FFI score: 14%). The patient had undergone physiotherapeutic sessions regularly (as confirmed by the therapist); NSAIDs have been taken only for 7 days after the diagnosis. Physical examination showed no tenderness, and the patient was able to bear full weight on the foot without discomfort. No adverse events have been reported during this time interval. A follow-up ultrasound demonstrated complete resolution of the plantar fascia rupture and no residual inflammation. He hadn't any limitations in practicing his routine sport activity. The patient was advised to continue strengthening exercises and use supportive footwear during high-impact activities to prevent future injuries.

## 4 Discussion

This case highlights the effectiveness of a conservative management approach in treating a healthy 35-year-old male patient who experienced a spontaneous plantar fascia rupture. We aimed to increase awareness of an uncommon yet impactful clinical condition, focusing on its diagnosis and management. This case underscores the importance of considering spontaneous rupture in the differential diagnosis of acute heel pain, even in the absence of typical risk factors, and supports the efficacy of non-surgical treatment modalities in managing such injuries. No guidelines or other unequivocal recommendations are available for the treatment of this condition (11); after performing a thorough examination of the evidence available in the Literature concerning plantar fascia rupture and plantar fasciopathies, we elaborated and proposed a novel integrated rehabilitative approach. This integrated rehabilitation approach was able to get a satisfying functional recovery in this case, as demonstrated by a significant reduction in VAS and FFI scores. The patient's recovery without surgical intervention underscores the potential benefits of non-invasive treatment strategies.

Conservative management of plantar fascia ruptures focuses on alleviating pain, reducing inflammation, promoting tissue healing, and gradually restoring function. This approach generally includes rest, immobilization, cryotherapy, physical therapy, and gradual reintroduction of weight-bearing activities. In the rehabilitation protocol, an adequate stretching program of the triceps surae structures has a prominent role, considering the relationship of the plantar fascia with the paratenon of the Achilles tendon (12), as confirmed in a cadaveric study performed by Stecco et al. in 2013 (13). Spontaneous rupture of the plantar fascia, defined as a rupture occurring in healthy patients with no prior foot risk factors, is rare with a few case reports reported in the literature (14–20). The rupture of the plantar fascia typically occurs due to chronic overuse, leading to microtears and degeneration, and predominantly affects athletes and individuals engaged in high-impact or repetitive activities. Factors contributing to its rupture include chronic plantar fasciitis, corticosteroid injections, obesity, and sudden increases in physical activity. Lee et al. examined the clinical characteristics and risk factors for plantar fascia rupture by comparing age, gender, affected side, BMI, visual analogue scale, previous treatments, degree of ankle dorsiflexion, the extent of activity, calcaneal pitch angle, heel alignment and the presence of a calcaneal spur. In their series, of these risk factors, only corticosteroid injections had a prominent role in the occurrence of a plantar fascia rupture (9). To prevent the risk of plantar fascia rupture, a therapeutic alternative to corticosteroid injections for the management of plantar fasciitis might be ultrasound-guided collagen injections, as reported by Corrado et al. in 2020 (21) but further studies are necessary. As emerged in the systematic review performed by Debus et al. in 2020 (including 78 studies with 124 patients) there are few available studies concerning the rupture of plantar fascia with poor data quality. Anyway, the current literature supports conservative management as an effective treatment for plantar fascia ruptures. As confirmed by the authors there is no standardized therapeutic approach. The maximum duration of immobilization of 3 weeks in a rigid walker with pain-adapted weight-bearing seems to be the most applied therapeutic protocol (11). A review by Mosca et al. highlighted that ruptures of the plantar fascia are more common in patients treated with local injections of steroids but are very rare in asymptomatic patients. According to the authors, surgical intervention might be necessary in severe, refractory, or chronic cases, while most patients respond well to conservative measures (although not standardized in the literature). The review underscored the importance of early diagnosis and appropriate management to ensure optimal recovery (10).

Our study has some limitations that should be addressed. First, this report discusses only one instance of spontaneous rupture of the plantar fascia, limiting the ability to generalize findings or draw broad conclusions. A larger series of cases or a comparative study would provide more robust data. Second, the follow-up period in this case was relatively short; extended follow-up would provide a more comprehensive understanding of the prognosis and effectiveness of the treatment. Third, the patient in this case was a healthy adult male with a specific lifestyle (recreational cyclist). The findings may not be generalizable to different populations, such as older adults, females, or individuals with comorbidities. Fourth, the assessment of pain and functional improvement relies heavily on patient-reported outcomes, which can be subjective. Objective measures such as functional tests could provide more reliable data. Finally, the case report does not delve deeply into potential etiological factors that could contribute to spontaneous rupture in the absence of identifiable risk factors. A discussion on possible underlying mechanisms, such as genetic predisposition or microscopic degenerative changes, would be valuable. By acknowledging these limitations, the report highlights areas for future research and emphasizes the need for more extensive studies to better understand and manage spontaneous plantar fascia ruptures.

## 5 Conclusion

This case report aims to increase awareness of spontaneous plantar fascia rupture, a rare and uncommon yet impactful injury, to keep in mind in the differential diagnosis. This case demonstrates that an integrated rehabilitative program can be highly effective in treating this pathological condition and lead to full functional recovery without the need for surgical intervention. The structured approach, including rest, immobilization, physiotherapy, and gradual return to activity, proved successful in this patient, offering a practical framework for clinicians managing similar cases. Continued research and documentation of such cases are essential to further validate and refine conservative treatment protocols for plantar fascia ruptures.

## Data Availability

The raw data supporting the conclusions of this article will be made available by the authors, without undue reservation.
